# Assessing Patient Perceptions and Experiences of Paracetamol in France: Infodemiology Study Using Social Media Data Mining

**DOI:** 10.2196/25049

**Published:** 2021-07-12

**Authors:** Stéphane Schück, Avesta Roustamal, Anaïs Gedik, Paméla Voillot, Pierre Foulquié, Catherine Penfornis, Bernard Job

**Affiliations:** 1 Kap Code Paris France; 2 Sanofi Paris France

**Keywords:** analgesic use, data mining, infodemiology, paracetamol, pharmacovigilance, social media, patient perception

## Abstract

**Background:**

Individuals frequently turning to social media to discuss medical conditions and medication, sharing their experiences and information and asking questions among themselves. These online discussions can provide valuable insights into individual perceptions of medical treatment, and increasingly, studies are focusing on the potential use of this information to improve health care management.

**Objective:**

The objective of this infodemiology study was to identify social media posts mentioning paracetamol-containing products to develop a better understanding of patients’ opinions and perceptions of the drug.

**Methods:**

Posts between January 2003 and March 2019 containing at least one mention of paracetamol were extracted from 18 French forums in May 2019 with the use of the Detec’t (Kap Code) web crawler. Posts were then analyzed using the automated Detec’t tool, which uses machine learning and text mining methods to inspect social media posts and extract relevant content. Posts were classified into groups: Paracetamol Only, Paracetamol and Opioids, Paracetamol and Others, and the Aggregate group.

**Results:**

Overall, 44,283 posts were analyzed from 20,883 different users. Post volume over the study period showed a peak in activity between 2009 and 2012, as well as a spike in 2017 in the Aggregate group. The number of posts tended to be higher during winter each year. Posts were made predominantly by women (14,897/20,883, 71.34%), with 12.00% (2507/20,883) made by men and 16.67% (3479/20,883) by individuals of unknown gender. The mean age of web users was 39 (SD 19) years. In the Aggregate group, pain was the most common medical concept discussed (22,257/37,863, 58.78%), and paracetamol risk was the most common discussion topic, addressed in 20.36% (8902/43,725) of posts. Doliprane was the most common medication mentioned (14,058/44,283, 31.74%) within the Aggregate group, and tramadol was the most commonly mentioned drug in combination with paracetamol in the Aggregate group (1038/19,587, 5.30%). The most common unapproved indication mentioned within the Paracetamol Only group was fatigue (190/616, with 16.32% positive for an unapproved indication), with reference to dependence made by 1.61% (136/8470) of the web users, accounting for 1.33% (171/12,843) of the posts in the Paracetamol Only group. Dependence mentions in the Paracetamol and Opioids group were provided by 6.94% (248/3576) of web users, accounting for 5.44% (342/6281) of total posts. Reference to overdose was made by 245 web users across 291 posts within the Paracetamol Only group. The most common potential adverse event detected was nausea (306/12843, 2.38%) within the Paracetamol Only group.

**Conclusions:**

The use of social media mining with the Detec’t tool provided valuable information on the perceptions and understanding of the web users, highlighting areas where providing more information for the general public on paracetamol, as well as other medications, may be of benefit.

## Introduction

### Background

Over-the-counter (OTC) medications are generally effective and associated with a well-documented safety profile when used as directed, making them convenient for managing ailments such as mild pain when medical consultation is not required [1]. The responsibility for the proper use of these medications falls on the individual using them, who may be guided by a pharmacist. While some information is available about an individual’s decision-making process when selecting medication, including demographic and social factors [2], more remains to be learned about issues that may affect an individual’s approach to taking OTC medications and whether additional information and guidance can further improve the safety and effectiveness of OTC medications. Areas of interest include symptom recognition (Do individuals understand their conditions?), self-selection (Why do they seek relief for their symptoms on their own?), active ingredients (Are the active ingredients known?), dosing (Do individuals distinguish between prescription regimens and use-as-needed instructions?), concomitant use warnings (Do individuals know the ingredients they need to heed because of warnings?), and when to stop the medication (Do individuals heed the maximum daily dose or duration of use warnings?).

Paracetamol is a well-established medication for pain management with a good safety profile when used as recommended, but increasingly, reports of inadequate use, including medication errors and misuse of the drug, have arisen [1,2]. In a study of OTC products containing paracetamol, 24% of adults confirmed they would take more than the recommended maximum dose within a 24-hour period; more than 20% of self-treating people struggled with dosing timing, such as taking another dose too soon; and 46% used more than one product with the same active ingredients [3]. Another study found many individuals do not routinely examine product label information for OTC pain relief, and more than 50% are unaware of the active ingredient [4]. The French National Agency for Medicines and Health Products Safety (ANSM) has therefore launched a campaign to raise awareness of the toxicity risks associated with the misuse of paracetamol [5].

Over the last decade, people have been increasingly using social media, including health forums, to communicate about and further understand all aspects of disease and treatment [6,7]. Compared with traditional medical data, social media can offer valuable insights into hundreds of thousands of individuals’ treatment management, including their opinions and concerns [8-10]. Topic mining in social media can document all the above aspects related to an individual’s experience with paracetamol and may also provide an adjunct to pharmacovigilance activities [11]. Different methods of social media data mining and topic modeling have been under investigation for use in pharmacovigilance studies to assist with postmarketing surveillance of drugs [12,13], and both the US Food and Drug Administration (FDA) and European Medicines Agency (EMA) are investigating the possibility of social media as a new data source to strengthen their activities regarding drug safety [14,15]. Detec’t (Kap Code, Paris, France) is an automated tool that uses artificial intelligence and text mining methods to analyze social media posts and extract relevant content. The Detec’t tool has previously been used to mine patient narratives from popular French forums for details such as potential adverse events (PAEs) and noncompliance, with a high level of specificity [11,13,16,17].

### Objective

Using the Detec’t tool, this infodemiology study investigated the opinions expressed by individuals on social media by identifying posts mentioning paracetamol-containing products.

## Methods

### Data Extraction

This was a retrospective infodemiology study assessing the contents of posts in social media about paracetamol products. A search was conducted in May 2019 to identify all posts made between January 2003 and March 2019, posts containing at least one keyword relating to paracetamol products were extracted from 18 general and specialized French social media channels (Multimedia Appendix 1) using the Detec’t web crawler as previously described [11,16]. Web scraping of the messages was performed depending on the HTML structure of each forum. Posts containing at least one keyword (Multimedia Appendix 2) were automatically retrieved with all the associated metadata, deidentified, and cleaned (signature and quote withdrawal).

### End Points

The primary end point was to identify the most frequent discussion themes in social media channels that mention paracetamol. The secondary end point was to identify PAEs for aggregated data analysis, including mentions of incorrect drug use.

### Preprocessing

The analysis corpus was cleaned after the removal of unrelated messages and posts in languages other than French. The posts were classified into Paracetamol Only, Paracetamol and Opioids, Paracetamol and Others, and Aggregate groups. The Paracetamol Only group included any post with only the predetermined 20 words associated with paracetamol; Paracetamol and Opioids included any drugs containing paracetamol and an opioid such as codeine or tramadol; Paracetamol and Others included drugs containing paracetamol and another nonopioid molecule, such as vitamin C; and the Aggregate group encompassed all 3 categories and contained all posts mentioned. Duplicate posts were removed; however, posts could be incorporated into more than one group if keywords relevant to multiple groups were identified.

### Processing and Statistical Analysis

#### Monitoring Algorithm

Postactivity volume information was identified with the use of a Markov chain–based algorithm, which clustered the data according to the time of posting [18,19]. The posts were separated into weak, moderate, or high activity; categories were defined not only regarding post volumes but also temporal dynamics, as permitted by Markov models. This allowed for the identification of unusual activity periods; for example, a marked increase in posts could correspond with a release of new safety-related information. A time series was created for the volume of posts per month, and data were corrected for periodic seasonality using locally estimated scatterplot smoothing (LOESS) regression on each seasonal subseries [20]. Estimation of the Hidden Markov Model (HMM) was performed using the expectation-maximization algorithm. The initial number of centroids was arbitrarily set to 3 in order to provide a simple categorization of posts’ volume observations. Observations were then clustered using a K-means clustering on the series trend and values, as well as the output of the HMM. Finally, smoothing of the clustering output was performed to obtain sequences of activity types.

#### Age and Gender

The gender of users was determined with the identification of specific expressions within the message (gendered past participles, adjectives, and names), as well as the application of a support vector machine (SVM) separating posts into female, male, or unknown. The model for gender identification was a linear SVM with a cost of 1. Age categories were also determined based on the use of specific expressions within the message.

#### Medical Concepts

The corpus of paracetamol posts that contained medical concepts was identified with the use of the Medical Dictionary for Regulatory Activities (MedDRA) version 15.0 and enriched with a web user vocabulary, as described elsewhere [16].

#### Discussion Topics

A topic model was applied to identify the themes addressed within the messages. These models are based on the hypothesis that each document in the corpus corresponds to a distribution of several topics. The modeled topics are probability distributions over the tokens (words or sequences of several adjacent words) found within the corpus. No prior assumption was made about the nature of topics present in the corpus. These models have been used previously to analyze health-related topics within web forums [21,22].

For this study, the correlated topic model based on the latent Dirichlet allocation was used [23]. The modeling of the studied corpus went through different preprocessing and cleaning steps so that the topic model could be applied. The model was estimated using a variational expectation-maximization algorithm. Topics, being probability distributions over tokens of the corpus of study, could be characterized by the highest per-topic probability tokens. Evaluating these probabilities through term-frequency inverse document frequency (TF-IDF) weighting allows the allocation of a higher importance to the topic specific tokens. In this case, the per-topic probability of a token was weighted by the inverse of the probabilities of this token in other topics. For each topic, tokens were ranked from highest- to lowest-weighted probabilities as per the TF-IDF value of their probability in this topic [24]. The first 15 tokens were designated as the set of characteristic tokens and used to name the topic. The analysis was performed using the structural topic model package [25] with R environment (version 3.5.2, R Foundation for Statistical Computing).

#### Top 10 Treatments

The top 10 treatments include the first 10 paracetamol-containing brands discussed most within the messages. The products mentioned were identified, and the occurrence of the medications in the posts was counted.

#### Drug-Intake Algorithm

A drug intake algorithm was used to determine whether each drug identified in the post had been taken by the web user. The messages were separated into individual sentences before specific variables were applied. Three distinct variables were used: lexical features (eg, intake and nonintake lexical fields), stylistic features (eg, proportion of exclamation marks, proportion of used pronouns), and syntactic structure features (eg, length of sentences, drug position). Analyzed content was scaled to avoid bias in the direction of the user who posted richer content (eg, number of posts or diversity of content). A random forest algorithm was applied to predict whether drug intake was expressed or not at the sentence level. Each sentence prediction was aggregated per post in order to have a global intake prediction per message.

#### Simultaneous Drug Consumption

Simultaneous drug consumption data were identified by assessing, per web user, the consumption of a paracetamol product (as per the list of predefined paracetamol-associated keywords) at the same time as another drug, detected with the use of the Detec’t medical product list (which contains approximately 2500 molecules and drugs). The drug intake algorithm was applied only to posts that mentioned paracetamol consumption, and the named-entity recognition technique allowed for the identification and labeling of the other drugs mentioned within the message.

#### Potential Adverse Events

Messages that included mention of drug intake were identified and then assessed for medical concepts (as determined with MedDRA). An SVM classifier, with a weighted radial SVM with cost 100 and gamma parameter 0.1, trained on an annotated gold standard [16], was then used to assess the messages to determine whether the post mentioning a paracetamol-containing product included a PAE. A machine learning model was used to train the SVM classifier; this used several clustering parameters, including the distance between the concept and the closest drug mention, the length of the message, and the number of times this concept was identified within the message. This clustering method is described elsewhere [16]. A manual review was performed by experts on one-third of the posts related to PAEs.

#### Unapproved Indications, Dependence, and Overdose

The analysis on incorrect use encompassed unapproved indications, dependence, and overdose. In order to assess incorrect drug use within adults only, data from use in children (aged younger than 15 years) were eliminated.

##### Unapproved Indications

Within each post expressing drug intake and medical concepts, stated drug use was assessed, manually filtered, and compared with the approved indications found within the product Summary of Product Characteristics (SmPC). These SmPC terms (from MedDRA) were used for each category of drug. Unapproved indications were defined as situations where a medical product was intentionally used for a purpose not in accordance with the terms of the marketing authorization. Posts containing medical concepts that were approved indications were excluded from the analysis corpus. The remainder were reviewed manually and for each unapproved indication selected by categories; 10% of messages were randomly sampled and annotated manually. The percentage shown represents the percentage of posts detected for the unapproved indication that were confirmed manually to have an unapproved indication.

##### Dependence

Dependence was determined by calculating a score through the BM25 [26] algorithm comparing a message and a reference pattern. The reference pattern was composed from a dependency lexical field, obtained from a sample of messages. The dependency lexical field contained words and expressions linked to drug dependency, which were manually reviewed by a group of experts.

All messages were divided into sentences containing the drug name and at least one of the words from the dependency lexical field. Each sentence within a message was then compared to the pattern reference using the BM25 score. The general score for each message was calculated by summation of each message. Higher scores revealed a similarity between the reference pattern and the message (eg, the higher the score, the more the sentence expressed dependency on a drug). To classify a message as discussing dependency, a similarity score threshold was chosen. Within this study, if a message had a similarity score equal to or greater than 40, it was identified as a message expressing dependency.

##### Overdose

The paracetamol overdose algorithm was based on linguistic rules used to construct an algorithm based on pattern matching. The algorithm highlighted overdose expressions by matching expressions constructed on linguistic and syntactic rules as “I[PRONOUN] take [VERB] 10[DIGIT]gr [DOSAGE EXPRESSION]” or lexical field related to an overdose as “overdose,” “addicted to paracetamol.” These expressions were obtained from manual reviews provided by a group of experts. The paracetamol dose contained in each unit of drugs (eg, pill or sachet) was reviewed manually and the quantity converted into units or grams. In the event of a discrepancy over the amount, a higher dose was assumed.

The paracetamol dose identified in the post was converted to a per-day dose by the algorithm and compared with the paracetamol daily dose threshold [27]. If the dosage expressed in a post was greater than the daily recommended dose of paracetamol, or if the post contained an expression from the lexical field of overdose, the post was classified as expressing an overdose. The overdose was calculated as a percent above the daily dose of the product in question. The algorithm was also able to detect lexical field words related to overdose. The number of users expressing a drug overdose was calculated by adding the number of users detected with the digits and dosage expressions above the daily dose, as well as the number of users detected with overdose lexical field expressions. Each user was counted once.

#### Ethical Considerations

Data collection and treatment followed the European Union General Data Protection Regulation. A privacy-by-design approach was adopted as retrieved posts were anonymized before being stored in the analysis corpus. Furthermore, all presented results were aggregated.

## Results

**Figure 1:**
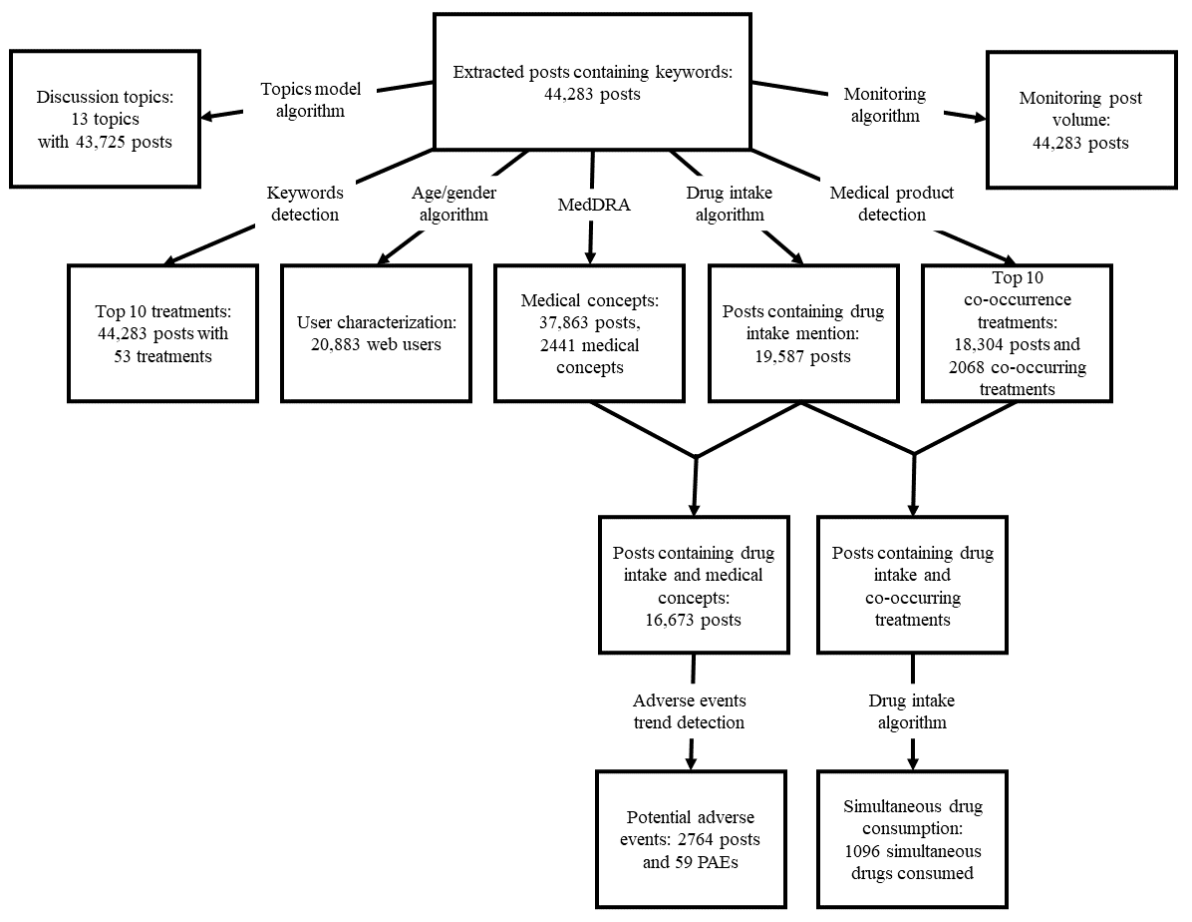
Categorization of posts containing paracetamol-related content. MedDRA: Medical Dictionary for Regulatory Activities; PAE: potential adverse event.

In May 2019, a search was conducted across 18 French forums (Multimedia Appendix 1) for posts made between January 2003 and March 2019. Figure 1 shows the distribution of posts associated with each category.

The Aggregate group contained 44,283 posts from 20,883 different users. Comparatively, the Paracetamol Only group had 33,196 messages (74.96% of total corpus) from 17,070 users (81.74% of total users); the Paracetamol and Opioids group had 14,733 (33.27%) messages, accounting for 6838 users (33.27%); and the Paracetamol and Others group had 1224 messages (2.76%) from 828 users (3.69%).

**Figure 2:**
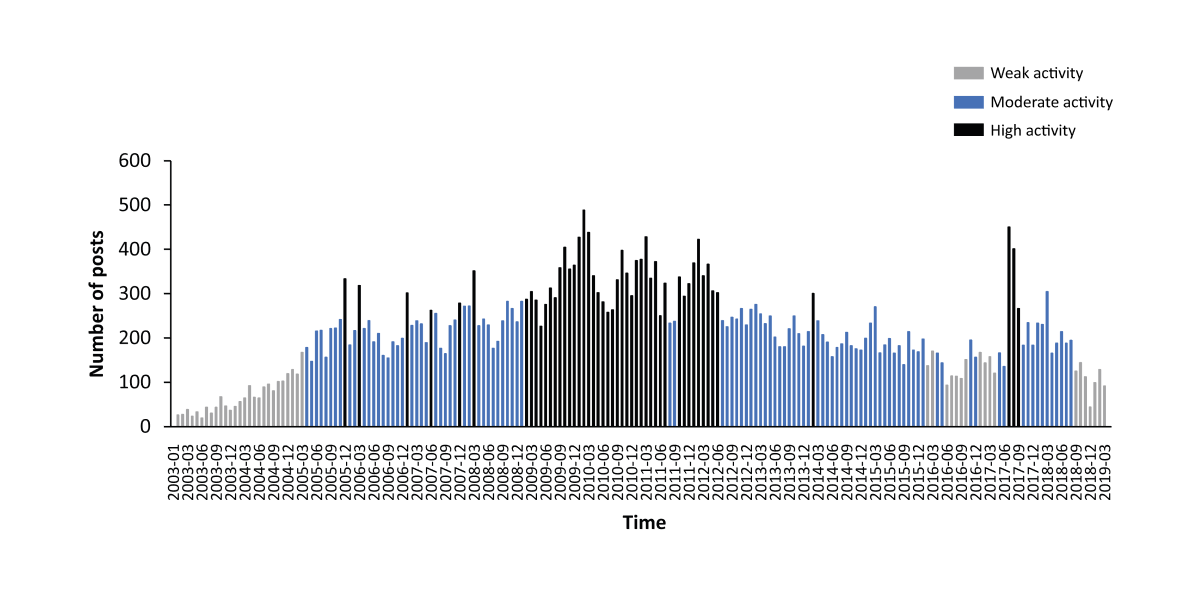
Volume of posts containing mention of paracetamol over time for the Aggregate group.

Figure 2 shows the variable activity seen in the post volume over the study period for the Aggregate group. A peak in postvolume activity was seen between 2009 and 2012, with a spike in activity seen in the middle of 2017. The number of posts tended to be higher during the winter and lower during the summer. Post activity for the individual groups is presented in Multimedia Appendices 3-5. The sociodemographic characteristics analysis shows that the posts were predominantly from women and the mean age of web users was 39 (SD 19) years (Table 1).

For the primary end point, pain was the most common medical concept discussed within the Paracetamol Only group with 15,310 posts (57.20%), as well as the Paracetamol and Opioids group with 6710 posts (65.41%; Table 2). Within the Paracetamol and Others group, nasopharyngitis was listed as the most common medical concept with 251 posts (30.0%), followed by pain (237 posts; 28.3%). Table 3 shows discussion topics identified across all 4 main groups, providing more specific information on the common threads web users were discussing. Paracetamol risk was the most common topic for the Aggregate group, addressed in 8902 posts (20.36%), followed by depression and suicide attempts found in 8063 posts (18.44%). Drug intake in everyday life, however, was the most common discussion topic for both the Paracetamol Only group (10,949 posts, 33.37%) and the Paracetamol and Opioids group (4235 posts, 29.10%). For the Paracetamol and Others group, cold medicine was the most common discussion point, addressed in 404 posts (34.38%).

**Table 1:** Sociodemographic characteristics of web users.

Characteristic			Paracetamol only (n=17,070), n (%)		Paracetamol and opioids (n=6838), n (%)		Paracetamol and others (n=828), n (%)		Aggregate^a^ (n=20,883), n (%)
**Gender**									
	Women	11,999 (70.29)		4985 (72.90)		563 (68.00)		14,897 (71.34)	
	Men	1962 (11.49)		821 (12.01)		105 (13.04)		2507 (12.00)	
	Unknown	3109 (18.21)		1032 (15.09)		160 (19.32)		3479 (16.66)	
	Total	17,070 (100.00)		6838 (100.00)		828 (100.00)		20,883 (100.00)	
**Age group (years)**									
	0-20	1767 (10.35)		593 (8.67)		74 (8.93)		2104 (10.08)	
	21-30	4565 (26.74)		1596 (23.34)		242 (29.23)		5454 (26.12)	
	31-40	4590 (26.89)		1795 (26.25)		234 (28.26)		5609 (26.86)	
	41-50	1874 (10.98)		936 (13.69)		81 (9.78)		2409 (11.54)	
	51-60	926 (5.42)		479 (7.00)		49 (5.91)		1226 (5.87)	
	60+	1982 (11.61)		1050 (15.36)		62 (7.49)		2611 (12.50)	
	Unknown	1366 (8.00)		389 (5.69)		86 (10.39)		1470 (7.04)	
	Total	17,070 (100.00)		6838 (100.00)		828 (100.00)		20,883 (100.00)	

^a^Aggregate users are the number of distinct users, and as such this column is not the sum of previous columns as a user may have posted in more than one other category.

**Table 2:** Top 10 medical concepts detected within the posts for each group.

Medical concept	Aggregate (n=37,863), n (%)	Paracetamol only (n=26,768), n (%)	Paracetamol and opioids (n=10,258), n (%)	Paracetamol and others (n=837), n (%)
Pain	22,257 (58.78)	15,310 (57.20)	6710 (65.41)	237 (28.32)
Fatigue	4279 (11.30)	2962 (11.07)	1244 (12.13)	73 (8.72)
Pyrexia	3564 (9.41)	3358 (12.54)	—^a^	61 (7.29)
Analgesic drug level	3193 (8.43)	1786 (6.67)	1295 (12.62)	112 (13.38)
Dependence	3177 (8.39)	1535 (5.73)	1586 (15.46)	56 (6.70)
Migraine	2840 (7.50)	1733 (6.47)	1032 (10.06)	75 (8.96)
Headache	2831 (7.48)	2091 (7.81)	672 (6.55)	68 (8.12)
Pregnancy	2322 (6.13)	1876 (7.01)	—	86 (10.27)
Adverse event	2022 (5.34)	—	733 (7.15)	—
Nausea	1898 (5.01)	1296 (4.84)	566 (5.51)	—
Vomiting	—	1335 (4.99)	—	—
Nasopharyngitis	—	—	—	251 (29.99)
Somnolence	—	—	—	55 (6.57)
Anxiety	—	—	559 (5.45)	—
Emotional distress	—	—	516 (5.03)	—

^a^Only the top 10 medical concepts are presented for each group. Instances where an entry is not listed does not mean that these medical concepts were not detected within the group.

**Table 3:** Most common discussion topics identified within the posts.

Topic			Posts, n (%)
**Aggregate (n=43,725)**			
	Paracetamol risk	8902 (20.36)	
	Depression and suicide attempt	8063 (18.44)	
	Drug intake in everyday life	7342 (16.79)	
	Children’s prescriptions	7320 (16.74)	
	Addiction	6554 (14.99)	
	Neuropathic pain	5604 (12.82)	
	Drug use during pregnancy	5481 (12.54)	
	Postsurgery pains	5456 (12.48)	
	Switch of medications	5357 (12.25)	
	Gynecology and paracetamol	5057 (11.57)	
	Posology and composition	4373 (10.00)	
	Migraine relief	3238 (7.41)	
	Alternative therapies	3021 (6.91)	
**Paracetamol only (n=32,807)**			
	Drug intake in everyday life	10,949 (33.37)	
	Gynecology and paracetamol	7085 (21.60)	
	Neuropathic pain	7025 (21.41)	
	Sharing information	5770 (17.59)	
	Addiction	5276 (16.08)	
	Children’s prescriptions	4947 (15.08)	
	Sleep disorders	4675 (14.25)	
	Postsurgery pains	4284 (13.06)	
	Advice for pain relief	3612 (11.01)	
	Migraine relief	3590 (10.94)	
	Care pathway	3534 (10.77)	
**Paracetamol and opioids (n=14,617)**			
	Drug intake in everyday life	4253 (29.10)	
	Addiction	3427 (23.45)	
	Incorrect opioid use	3416 (23.37)	
	Migraine relief	3195 (21.86)	
	Neuropathic pain	3048 (20.85)	
	Depression and suicide attempt	3022 (20.67)	
	Chronic pain	2530 (17.31)	
	Postsurgery pain	2480 (16.97)	
	Acute pain treatment (gynecologic, toothaches)	2457 (16.81)	
	Posology and composition	282 (1.93)	
**Paracetamol and others (n=1175)**			
	Cold medicines	404 (34.38)	
	Ear, nose, and throat problems	371 (31.57)	
	Drug use during pregnancy	331 (28.17)	
	Adverse events	211 (17.96)	
	Migraine relief	180 (15.32)	
	Dizziness sensations	138 (11.74)	
	Self-prescriptions	127 (10.81)	
	Therapeutic options	126 (10.72)	
	Posology and composition	71 (6.04)	
	Drugs during breastfeeding	26 (2.21)	

The analysis displaying the top 10 treatments mentioned in the posts identified Doliprane as the most common medication with 14,058 posts (31.74%), followed by Dafalgan with 4812 posts (10.86%), and Ixprim with 4344 posts (9.80%). Assessing simultaneous drug consumption, tramadol was the drug most commonly mentioned in combination with paracetamol across all groups except Paracetamol and Others; in the Aggregate group, tramadol accounted for 1038 posts (5.30% of all posts suggesting a paracetamol drug intake), followed by ibuprofen (601 posts, 3.07%). In the Paracetamol and Others group, caffeine was most commonly consumed with paracetamol, accounting for 65 posts (14.04%).

Assessing posts containing possible incorrect use, as per the secondary end points, the algorithm for unapproved indication detection identified 190 posts associated with fatigue, the most common unapproved indication mentioned within the Paracetamol Only group. Following a manual review, 16.32% of these posts were found to be positive for unapproved indications, with the remainder of posts associated with fatigue describing fatigue as a symptom or an effect of the drug intake. Dependence was detected in 148 posts, of which 14.19% were manually validated for an unapproved indication. Within the Paracetamol and Opioids group, dependence was detected in 238 posts, of which 66.81% were manually validated for an unapproved indication. This was followed by fatigue, which was detected in 110 posts and manually validated in 9.10%.

Within the Paracetamol Only group, posts with reference to dependence (as identified with the use of the BM25 algorithm), were made by 1.61% of the web users, accounting for 1.33% of the posts. Comparatively, dependence mentions in the Paracetamol and Opioids group were provided by 6.94% of web users, accounting for 5.44% of total posts.

Reference to overdose was made by 245 web users across 291 posts within the Paracetamol Only group. The most referenced drug was Doliprane, mentioned by 88 web users. For the Paracetamol and Opioids group, reference to overdose was made by 128 web users across 177 posts, and Codoliprane was the most referenced drug, mentioned by 67 web users. Some posts referenced overdoses at up to 500% above the recommended daily dose.

The top 10 PAE trends, which include mention of drug intake, are displayed in Table 4. For the Paracetamol Only group, the most common event detected was nausea, mentioned in 2.38% of posts detected by the SVM classifier, followed by vomiting (2.27%) and hypersensitivity (1.24%). For the Paracetamol and Opioids group, the most common event was dependence (7.93% of posts), followed by somnolence (2.55%).

**Table 4:** Top 10 potential adverse event trends detected within the posts.

Potential adverse event trends		Posts, n (%)^a^
**Paracetamol only (n=12,843)**		
	Nausea	306 (2.38)
	Vomiting	292 (2.27)
	Hypersensitivity	159 (1.24)
	Substance abuse	141 (1.10)
	Dizziness	107 (0.83)
	Feeling abnormal	73 (0.57)
	Malaise	68 (0.53)
	Overdose	64 (0.50)
	Chills	62 (0.48)
	Feeling jittery	59 (0.46)
**Paracetamol and opioids (n=6281)**		
	Dependence	498 (7.93)
	Somnolence	160 (2.55)
	Substance abuse	125 (1.99)
	Insomnia	103 (1.64)
	Dizziness	101 (1.61)
	Substance-induced psychotic disorder	74 (1.18)
	Withdrawal syndrome	71 (1.13)
	Feeling abnormal	56 (0.89)
	Constipation	47 (0.75)
	Euphoric mood	40 (0.64)

^a^Percentage does not represent the frequency of occurrence in treated patients, but rather the percentage of social media posts found within the drug intake and medical concepts cohorts, in which mention of a given potential adverse event was detected with the adverse events detection algorithm.

## Discussion

### Principal Findings

This study aimed to identify the most frequent themes related to the paracetamol products being discussed within 18 French forums. The data demonstrated that many individuals use social media to converse regarding paracetamol-containing products, discussing drug efficacy, safety, and more. The Detec’t tool captured the information within these discussions and separated it, creating categories to allow for the analysis of particular areas of interest.

The data acquired within this study provide valuable information on web-based discussions involving paracetamol, allowing a better understanding of the needs and concerns of individuals taking or considering taking paracetamol. The demographic analysis within this study appears to concur with previous findings. Of the posts made by people of a known gender, there was more content from women than men, demonstrating a potential bias in the web users. This has been noted within other studies, and it is recognized that women are generally more likely to frequent web forums to discuss health-related information than men [28,29]. The mean age of web users was 39 years, slightly younger than in findings from a US study, which found that the majority of individuals using drug review websites were aged 45 to 64 years [29].

A higher volume of posts was generally seen in colder months than warmer months, possibly linked to cold weather causing an increase or worsening in the prevalence of common illnesses that cause pain or fever [30,31]. The spike in post activity seen within the Paracetamol Only and Paracetamol and Opioids groups between 2009 and 2012 corresponds with the withdrawal of dextropropoxyphene-containing medicines from the market [32,33]. Another spike in post activity seen in 2017 likely corresponds to a change in drug regulations, with all opioid-containing medication requiring a prescription from July 2017 onward [34]. Some of the content within these posts referenced finding alternative products for recreational use, as well as methods for separating paracetamol from opioids in combined medication, supporting the notion that the prescription mandate spike was associated with the discussions around opioid addiction. This is supported by comments discussing addiction and fear of not being able to access the drug, as well as alternative treatments. Given that pain and pyrexia are indications for which paracetamol may be used [27], it was not surprising that pain was the most common medical concept discussed across all posts and pyrexia was the third most common. Fatigue, the second most common medical concept, was often mentioned in relation to feeling unwell due to illness or sleep disorders caused by pain or fever.

The simultaneous drug consumption analysis found that taking paracetamol in combination with other medications or molecules was common. A study conducted in France found that approximately 23% of individuals were dispensed paracetamol in combination with another agent [2]. Combinations such as including tramadol with paracetamol are common. The different mechanisms of action of the paracetamol and weak opioids are believed to provide improved analgesic efficacy [35]. As an OTC medication, many individuals may not consider consulting a clinician or pharmacist on the risks of taking different OTC drugs and may be unaware that paracetamol is contained in various OTC drugs. This finding highlights the need for information targeting the general public, addressing the possible consequences of taking paracetamol concomitantly with other medications.

In addition to providing general information on paracetamol use, the Detec’t tool was also able to identify messages that indicated potential incorrect use of paracetamol, including exceeding the maximum daily recommended dose and dependence. This provides an insight into the views of the individuals who take paracetamol and highlights areas where increased consumer awareness of the dangers of incorrect use could be improved. The number of references to incorrect use suggests that further educational efforts may be required. Many individuals may be unaware of the potential toxicity associated with overdose, and the warning message recently added on the boxes in France should increase consumer awareness of this risk [5,36]. As Doliprane is one of the most popular brands of paracetamol mentioned within the top 10 treatments, it was unsurprising that it was the most popular brand mentioned within the analysis on overdose.

Underreporting of adverse drug events in real-world use has previously been documented, and a 2017 study confirmed this finding, demonstrating that for biologics and drugs with a narrow therapeutic index, approximately 20% to 33% of the minimum number of expected serious events were reported [37]. Social media mining has been used in studies to better understand the consumer mindset and may be able to assist with pharmacovigilance. A study conducted on the public opinion of women with inflammatory bowel disease (IBD) found 1818 posts relating to reproductive concerns for women taking medication for IBD. However, while the women had attributed a risk of reproductive problems to the medication, the condition itself can cause reproductive problems. This valuable information can be used to assist health care professionals to preemptively address these concerns in women taking IBD medication [9]. Within another study investigating methylphenidate for treatment of attention-deficit/hyperactivity disorder, within 3443 social media posts published between 2007 and 2016, 61 adverse events (AEs) were detected along with cases of misuse [13]. A study investigating the use of social media for toxicovigilance purposes found that within 6400 Twitter posts, tweets containing abuse signals were significantly higher for the 3 prescription medications—Adderall (22.6%), quetiapine (5.1%), and oxycodone (12.3%)—compared with metformin (0.3%), a control medication [38]. The WEB-RADAR (Recognizing Adverse Drug Reactions) project [39] investigated the value of data mining Facebook and Twitter for safety signal and AE detection compared with Vigibase [40]. While these social media platforms did not provide valuable pharmacovigilance information, it was suggested that more specialized social media platforms could enrich traditional signal detection, particularly for areas such as pregnancy and drug abuse/misuse [39].

Data mining of social media conversations regarding medication use allows a better understanding of individuals’ perceptions and knowledge about medications, often uncovering conversations that are not conducted within the health care setting. These findings may be used to improve health care by preemptively addressing areas of concern and also demonstrate that more easily accessible health care information for the general public would be beneficial. Paracetamol risk, depression, and suicide attempt were the most common discussion topics, suggesting that due to ease of availability, paracetamol consumption in toxic doses for suicidal purposes may be an issue, in line with previously published studies [41,42]. Additionally, discussion topics such as drug use during pregnancy and prescriptions for children indicate that targeting information on the safe and effective use of paracetamol to the public may be beneficial in order to forestall the possible spread of misinformation through online forums. Morbidity and mortality related to self-poisoning can be significantly reduced when a limit is imposed on the sale of paracetamol in a single purchase, suggesting that positive initiatives may assist in protecting the public [43].

A public consultation was launched by ANSM in 2018 with the aim of reducing the number of paracetamol-associated overdoses and medication errors [5]. One of the solutions identified by health authorities was to add a warning message to the product label regarding the risk of overdose with paracetamol, a measure implemented after the end of our study. With the additional information provided by the health authorities, it is hoped that individuals who use paracetamol will have a positive shift in behavior with the increased awareness of the drug’s risk [5]. It may be interesting to repeat this study to determine if any changes in how paracetamol is discussed on social media platforms since the introduction of these measures by ANSM can be detected. Such measures should be tailored to the awareness level of the population regarding appropriate use and adverse effects of paracetamol. Whereas a cross-sectional survey conducted in Saudi Arabia showed overall awareness of correct use of paracetamol to be low [44], the advantage of using web-based information collection includes the ability to gather information from individuals who might not otherwise take part in studies [10], as well as the ability to conduct global analyses with real-time collection from a broad sociodemographic range [9,45-47].

### Limitations

The statistical algorithms used have been developed for use on large pharmacovigilance databases, opening the possibility of conducting very large pharmacovigilance studies with relative ease. While this method has advantages over traditional information gathering, there are also limitations, including the fact that this is based on the web user’s declaration.

The majority of the posts were made by women, and this potential bias should be considered because women have been shown to display different behavior when searching for information online compared with men and are more likely to consult more sources and value content that is easier to grasp, whereas men tend to prefer a more comprehensive or accurate source [48].

The findings presented are data generated through machine learning predictions. Analyzing the unapproved indications identified within posts containing paracetamol references (eg, the indications) may therefore be viewed as pertaining to paracetamol (ie, linked with paracetamol), or alternatively the data may be unrelated. The style of language used by the web user, including metaphors, slang, or euphemisms, can prove challenging to code as well, although some alternative drug names were included, as shown in Multimedia Appendix 1. Of note, the potential AEs reported are not a reflection of the actual number of AEs; instead, they represent the proportion of posts describing a medical concept that was found to be associated with a potential AE due to drug intake as determined by an algorithm. In addition, some mentions of possible AEs may be confused with drug indications or questions about a possible risk of AE, which would be more likely to be differentiated with additional steps in the analysis, such as human review. Generally, subtleties within the posts may be missed with the use of the machine learning model. Despite these possible sources of bias, the Detec’t web-based information collection system used within this study has been previously validated in studies investigating individuals’ opinions on drugs via social media and has been found to have good specificity when identifying signals of disproportionate reporting within French medical forums instead of the traditional reporting system [11], including being over 95% accurate in removing posts containing nonadverse drug reactions [16]. However, it should be noted that the results we present are specific for this study and search parameters, so it is not possible to guarantee the algorithm was as successful in removing false positives or negatives. Additionally, the results are specific to France and may not be generalizable to other French-speaking countries or regions due to the cultural sensitivity of some topics.

### Conclusion

The use of social media mining with the Detec’t tool provided valuable information on the perceptions and understanding of the web users, highlighting areas where providing more information for the general public on paracetamol, as well as other medications, may be of benefit.

## Appendix

Multimedia Appendix 1 Forums used for postextraction.

Multimedia Appendix 2 List of keywords used for the postextraction categorized by drug composition.

Multimedia Appendix 3 Volume of posts containing mention of paracetamol over time for the Paracetamol Only group.

Multimedia Appendix 4 Volume of posts containing mention of paracetamol over time for the Paracetamol and Opioids group.

Multimedia Appendix 5 Volume of posts containing mention of paracetamol over time for the Paracetamol and Others group.

## Abbreviations

AE: adverse event
ANSM: Agence Nationale de Sécurité du Médicament et des Produits de Santé (French National Agency for Medicines and Health Products Safety)
EMA: European Medicines Agency
FDA: Food and Drug Administration
HMM: Hidden Markov Model
IBD: inflammatory bowel disease
LOESS: locally estimated scatterplot smoothing
MedDRA: Medical Dictionary for Regulatory Activities
OTC: over-the-counter
PAE: potential adverse event
SmPC: Summary of Product Characteristics
SVM: support vector machine
TF-IDF: term-frequency inverse document frequency
WEB-RADR: Recognizing Adverse Drug Reactions
