# Supplementary material for: Assessing Patient Perceptions and Experiences of Paracetamol in France: Infodemiology Study Using Social Media Data Mining
Source: J Med Internet Res. 2021 Jul 12;23(7):e25049. doi: 10.2196/25049 (PMC8314157; doi:10.2196/25049)
Supplement: Multimedia Appendix 1 [file jmir_v23i7e25049_app1.docx]

Forums used for post extraction.

| Name of forum | Website | Forum topic |
| --- | --- | --- |
| Atoute | www.forum.atoute.org | Medical |
| Aufeminin | www.aufeminin.com | General lifestyle |
| Doctissimo | www.forum.doctissimo.fr | Medical |
| Forum ados | www.forum.ados.fr | General lifestyle |
| Futura-Sciences | www.forums.futura-sciences.com | General lifestyle |
| Laxophobie | www.laxophobie.fr | Medical |
| Les Impatientes | www.lesimpatientes.com | Medical |
| Ligue contre le cancer | www.ligue-cancer.net | Medical |
| Lymphome Espoir | www.francelymphomeespoir.fr | Medical |
| Magicmaman | www.forum.magicmaman.com | General lifestyle |
| Maman pour la vie | www.mamanpourlavie.com | General lifestyle |
| MeaMedica | www.meamedica.fr | Medical |
| Onmeda | www.onmeda.fr | Medical |
| PsychoActif | www.psychoactif.org | Medical |
| Psychologies | www.forum.psychologies.com | General lifestyle |
| Santé médecine | www.sante-medecine.journaldesfemmes.fr | Medical |
| Thyroïde | www.forum-thyroide.net | Medical |
| Vulgaris Médical | www.vulgaris-medical.com/forum-sante | Medical |
