# Supplementary material for: Assessing Patient Perceptions and Experiences of Paracetamol in France: Infodemiology Study Using Social Media Data Mining
Source: J Med Internet Res. 2021 Jul 12;23(7):e25049. doi: 10.2196/25049 (PMC8314157; doi:10.2196/25049)
Supplement: Multimedia Appendix 2 [file jmir_v23i7e25049_app2.docx]

List of keywords used for the post extraction categorized by drug composition.

| Paracetamol Only | Paracetamol and Opioids | Paracetamol and Others |
| --- | --- | --- |
| Acetaminophene | Algicalm | Actifed (Actiphed; actifede) |
| Algodol (Algodole) | Algisedal | Actifedsign (Actifed Sign) |
| Claradol (Claradole) | Claradol Codeine | Actron |
| Dafalgan (daphalgan) | Codoliprane (Codolipran; codolipranne; codolliprane) | Algodol Cafeine |
| Dafalgan Pediatrique | Compralgyl | Algotropyl |
| Dafalganhop | Dafalgan Codeine | Cefaline Hauth |
| Doliprane | Doliprane Codeine | Claradol Cafeine |
| Dolipranecaps (Doliprane Caps) | Efferalgan Codeine | Clarix Etat Grippal |
| Dolipraneliquiz (Doliprane Liquiz) | Gaosedal Codeine | Coquelusedal Paracetamol |
| Dolipraneorodoz (Doliprane Orodoz) | Ixprim (Ixprime) | Doli Etat Grippal |
| Dolipranetabs (Doliprane Tabs) | Izalgi | DolipranevitamineC (Doliprane Vitamine C) |
| Dolko | Klipal Codeine | Dolirhume |
| Dolstic | Lamaline (Lammaline; Lamalin) | Dolirhumepro (Dolirhume Pro) |
| Efferalgan (Eferalgan) | Lindilane | Drill Rhume |
| Efferalganmed | Migralgine | Efferalgan Vitamine C |
| Geluprane | Novacetol | Excedrinil |
| Panadol | Paracetamol Codeine | Fervex (Fairevex; Fairvex; Fervexe) |
| Paracetamol (paracetamole; paracetamolle; paracetamaule) | Prontalgine | Fervexrhume |
| Paralyoc | Zaldiar | Flustimex |
| Perfalgan |  | Humexlib Etat Grippal |
|  |  | Humexlib |
|  |  | Humex Rhume (Hummex; Umex) |
|  |  | Rhumagrip |
|  |  | Theinol |
|  |  | Trophires Compose |

Names in brackets are alternate forms of the drug name considered during the data extraction as substitutes for the correct drug names.
