# Supplementary figures and images for: Assessing Patient Perceptions and Experiences of Paracetamol in France: Infodemiology Study Using Social Media Data Mining
Source: J Med Internet Res. 2021 Jul 12;23(7):e25049. doi: 10.2196/25049 (PMC8314157; doi:10.2196/25049)

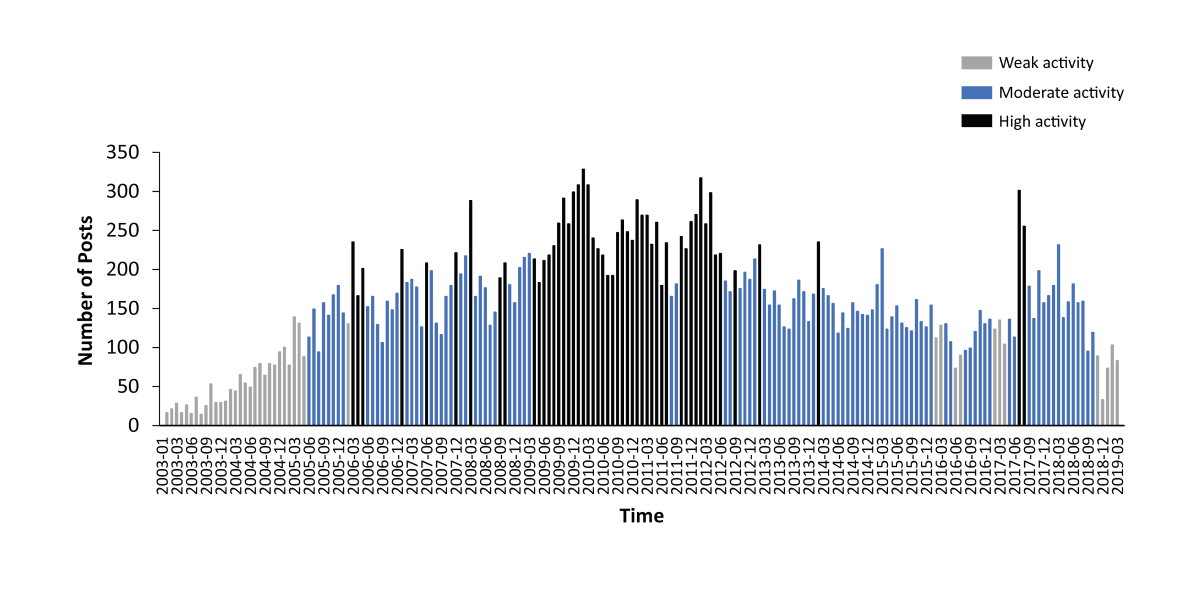

Supplement: Multimedia Appendix 3 [file jmir_v23i7e25049_app3.png]

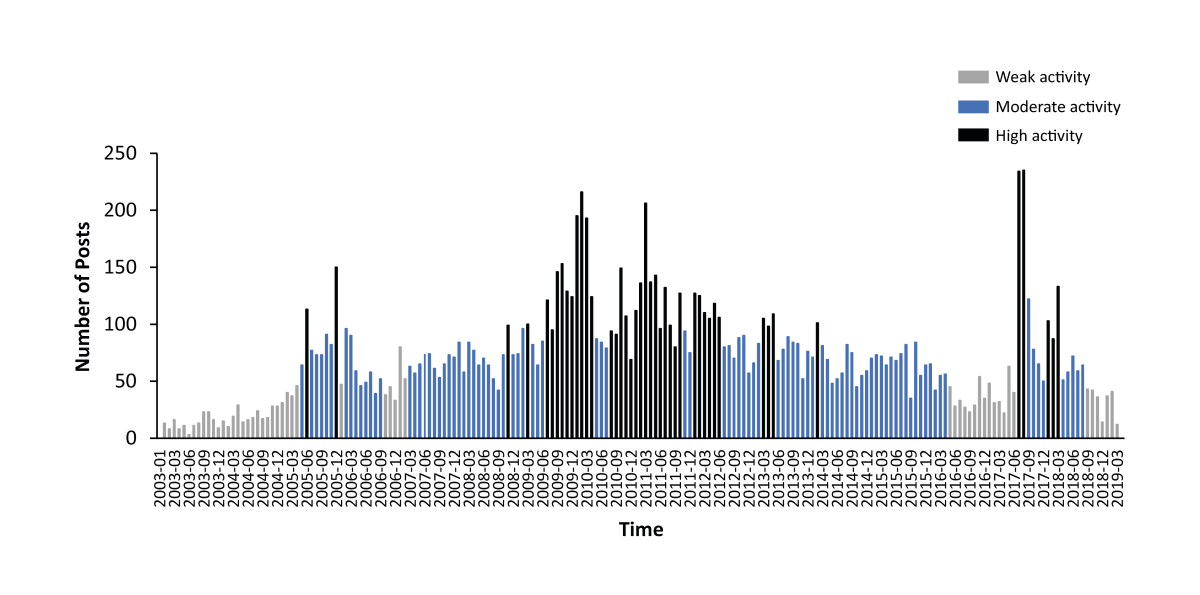

Supplement: Multimedia Appendix 4 [file jmir_v23i7e25049_app4.png]

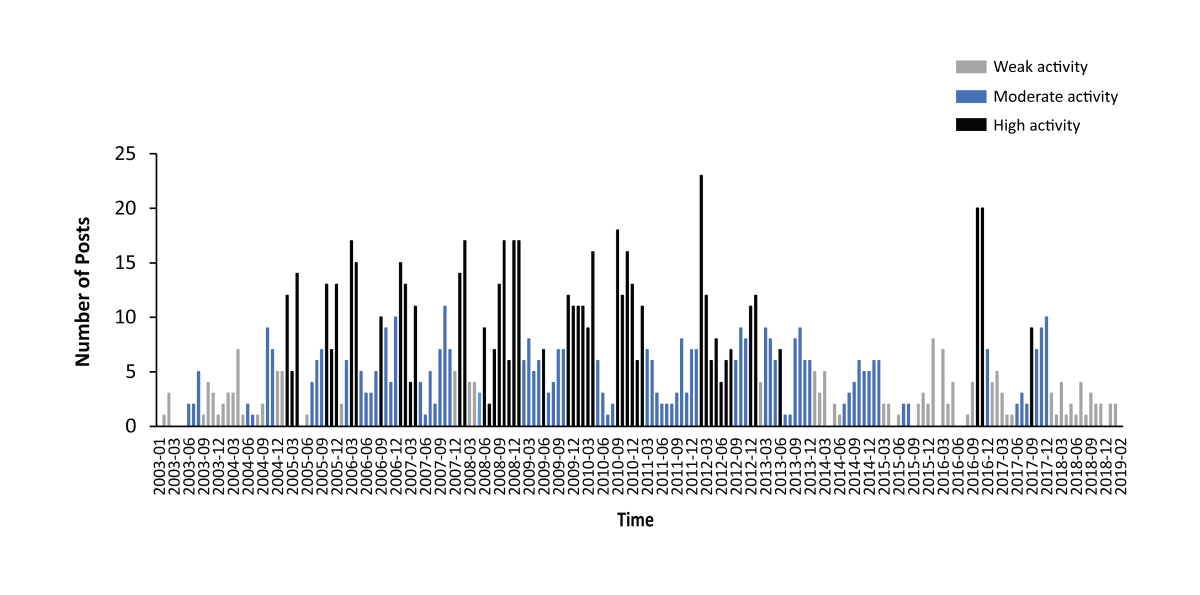

Supplement: Multimedia Appendix 5 [file jmir_v23i7e25049_app5.png]
